# Influence of Fat Content and Bioprotective Cultures on the Survival of Foodborne Pathogens in Beef Meatballs

**DOI:** 10.1002/fsn3.72260

**Published:** 2026-08-17

**Authors:** Enise Begüm Göçmez, Tevhide Elif Güner, Hakan Tavsanli, Osman İrfan İlhak

**Affiliations:** ^1^ Department of Food Processing, Kiraz Vocational School Dokuz Eylül University İzmir Türkiye; ^2^ Department of Food Hygiene and Technology, Faculty of Veterinary Medicine Balıkesir University Balıkesir Türkiye; ^3^ Department of Veterinary Public Health, Faculty of Veterinary Medicine Balıkesir University Balıkesir Türkiye

**Keywords:** beef meatballs, bioprotective cultures, *E. coli O157:H7*, fat content, *L. monocytogenes*, *Salmonella* spp.

## Abstract

This study evaluated the effects of fat content (low vs. regular) and bioprotective cultures ( *Pediococcus acidilactici* , *Latilactobacillus sakei*, and *Lactiplantibacillus plantarum*) on the survival of *Salmonella* spp., *Escherichia coli* O157:H7, and *Listeria monocytogenes* in beef meatballs stored at 3°C and 7°C. The addition of bioprotective cultures generally lowered pH values compared with untreated controls (*p* < 0.05). Differences in fat content were not consistently associated with changes in the survival of *Salmonella* spp. or *E. coli* O157:H7. However, in regular‐fat meatballs, the presence of bioprotective cultures resulted in notable pathogen reductions—reaching up to 1.6–2.4 log_10_ CFU/g—at both storage temperatures (*p* < 0.05). In contrast, the inhibitory effect against *L. monocytogenes* was less pronounced in low‐fat formulations. Growth of *Pseudomonas* spp. was slower in regular‐fat meatballs stored at 3°C. Overall, the results suggest that the combined use of bioprotective cultures and fat content may influence microbial control in beef meatballs, particularly under refrigerated storage conditions.

## Graphical abstract

Influence of Fat Content and Bioprotective Cultures on the Survival of Foodborne Pathogens in Beef Meatballs During Refrigerated Storage: Low‐fat and regular‐fat meatballs were prepared with or without bioprotective cultures and inoculated with *Salmonella*, *Listeria monocytogenes* , and *Escherichia coli* O157:H7. Samples were stored at 3°C and 7°C and microbiologically analyzed on days 0, 3, 5, and 7. Bioprotective cultures reduced pathogen survival, while regular‐fat meatballs exhibited stronger antimicrobial effects at 3°C, suggesting that fat content may enhance bioprotective culture activity.

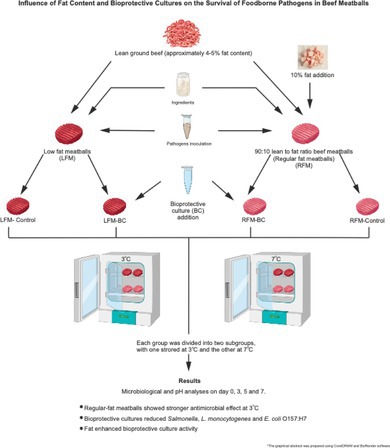

## 1 Introduction

Foodborne pathogens such as *Salmonella* spp., *Escherichia coli* O157:H7, and *Listeria monocytogenes* continue to pose a major public health concern, with meat products being among the primary vehicles of contamination. Ground beef is particularly susceptible due to its high water activity, near‐neutral pH, and increased surface area, which collectively favor microbial growth (Marcelli et al. 2024). Several outbreaks have been linked to contaminated ground beef, underscoring the need for effective control strategies (Reis et al. 2024).

Bioprotective lactic acid bacteria (LAB) have gained attention as natural antimicrobials for meat products (Barcenilla et al. 2022). Species such as *Pediococcus acidilactici* , *Latilactobacillus sakei*, and *Lactiplantibacillus plantarum* can inhibit a wide range of pathogens through acidification, bacteriocin production, and competitive exclusion (Singh et al. 2025; İncili et al. 2025). Their ability to grow or remain metabolically active at refrigeration temperatures makes them promising candidates for enhancing the microbial stability of fresh meat (Carneiro et al. 2024; Göçmez and İlhak 2025). However, the efficacy of LAB as bioprotective cultures can vary substantially according to the food matrix, target microorganism, and storage conditions. In particular, factors such as temperature fluctuations, pH, and interactions with the native microbiota strongly influence antimicrobial performance (Barcenilla et al. 2022).

Although refrigeration below 4°C is generally recommended for fresh meat products, temperature abuse during retail display, transportation, or domestic storage remains common (Ovca et al. 2021). Under such circumstances, the performance and reliability of LAB‐based bioprotective cultures become particularly important.

Fat content plays a key role in both the nutritional and technological properties of ground meat. Increased consumer demand for leaner meat products has encouraged the production of low‐fat formulations, while regular‐fat or high‐fat products remain widely consumed due to their superior flavor, juiciness, and texture (Cardona et al. 2020). Typical fat levels in ground beef vary considerably across regions and market categories, ranging from approximately 5% in lean products to 15% in regular products, depending on customer demand (Wang et al. 2020; Çimen and Çiçek 2021). Because fat may influence microbial stability, water distribution, and the diffusion of antimicrobial metabolites, its interaction with bioprotective cultures is of particular interest. Despite extensive research on LAB applications in meat preservation, little is known about how fat level may modulate the antimicrobial performance of LAB (Xu et al. 2023), especially under varying storage temperatures.

This study addresses this gap by formulating beef meatballs with regular‐fat (12%–13%) and low‐fat (4%–5%) beef, with and without bioprotective cultures. Products were stored under optimal refrigeration (3°C ± 1°C) or temperature abuse conditions (7°C ± 1°C) and challenged with *Salmonella* spp., *L. monocytogenes* , and *E. coli* O157:H7. The objective was to evaluate how fat content and cold‐chain integrity influence the effectiveness of LAB‐based bioprotective cultures in suppressing major foodborne pathogens in beef meatball.

## 2 Materials and Methods

### 2.1 Bacterial Cultures and Inoculum Preparation

In this study, a bioprotective mixture consisting of *Pediococcus acidilactici* (Bactoferm B‐LC‐20, Chr. Hansen), *Latilactobacillus sakei* (Bactoferm B‐FM, Chr. Hansen), and *Lactiplantibacillus plantarum* (DSMZ 16627, Bioferm) was used. The starter culture Bactoferm B‐FM (Chr. Hansen) utilized in this work comprised a combination of *L. sakei* and *Staphylococcus xylosus* strains. To initiate the bacterial growth, a 20‐h incubation of the strains was carried out at 30°C in de Man, Rogosa, and Sharpe (MRS, Biokar, Beauvais, France) broth. Following the activation phase, individual colonies were obtained by streaking the cultures onto MRS agar medium. Gram staining was applied to characterize the isolates, and colonies exhibiting bacillary morphology, corresponding to *L. sakei* , were selected. These isolates were further purified and used in subsequent analyses.

Individual bacterial strains were grown in 10 mL volumes of MRS broth for 18–20 h at a temperature of 30°C. The resulting cultures were then subjected to cold centrifugation to remove the supernatant. To obtain the final inocula, the remaining bacterial pellets were redissolved using 0.1% peptone solution. Equal volumes of *Pediococcus acidilactici* , *Latilactobacillus sakei*, and *Lactiplantibacillus plantarum* were combined to obtain approximately 9 log_10_ CFU/mL, as determined by enumeration on MRS agar, and were applied to each meatball formulation at a volume of 5 mL.

Pathogen cocktails were prepared using three strains each of *Salmonella* spp. (S. Typhimurium ATCC 14028, NCTC 12416, and S. Enteritidis ATCC 13076), *L. monocytogenes* (ATCC 7644, 13932, and N7144), and *E. coli* O157:H7 (ATCC 43984, 43895, and 35150). For the cultivation of individual strains, 10 mL volumes of sterile Tryptic Soy Broth (TSB; Merck, Germany) were utilized at 30°C for a duration of 18–20 h. Upon completion of the incubation period, cell harvesting was performed via centrifugation, followed by the reconstitution of the resulting pellets in 0.1% peptone solution. For each pathogen, three strains belonging to the same species were combined in equal proportions to prepare pathogen‐specific inocula (10 mL total volume). One milliliter of each inoculum was serially diluted and plated on selective agar media, then incubated at 37°C for 24–48 h for enumeration. The initial inoculum concentration to be added to meatballs was adjusted to approximately 7.5–8.0 log_10_ CFU/mL.

### 2.2 Preparation of Meatball Formulations

Lean beef and subcutaneous beef fat were purchased from a local butcher. The “top round” part of the carcass was used for mincing. Both meat and fat were ground separately twice through a 4.5 mm plate using an industrial meat grinder. The fat levels selected in this study were intended to reflect meatballs made from low‐fat to medium‐fat ground meat produced by small‐scale producers or local butchers, rather than high‐fat commercial products.

**FIGURE 1:**
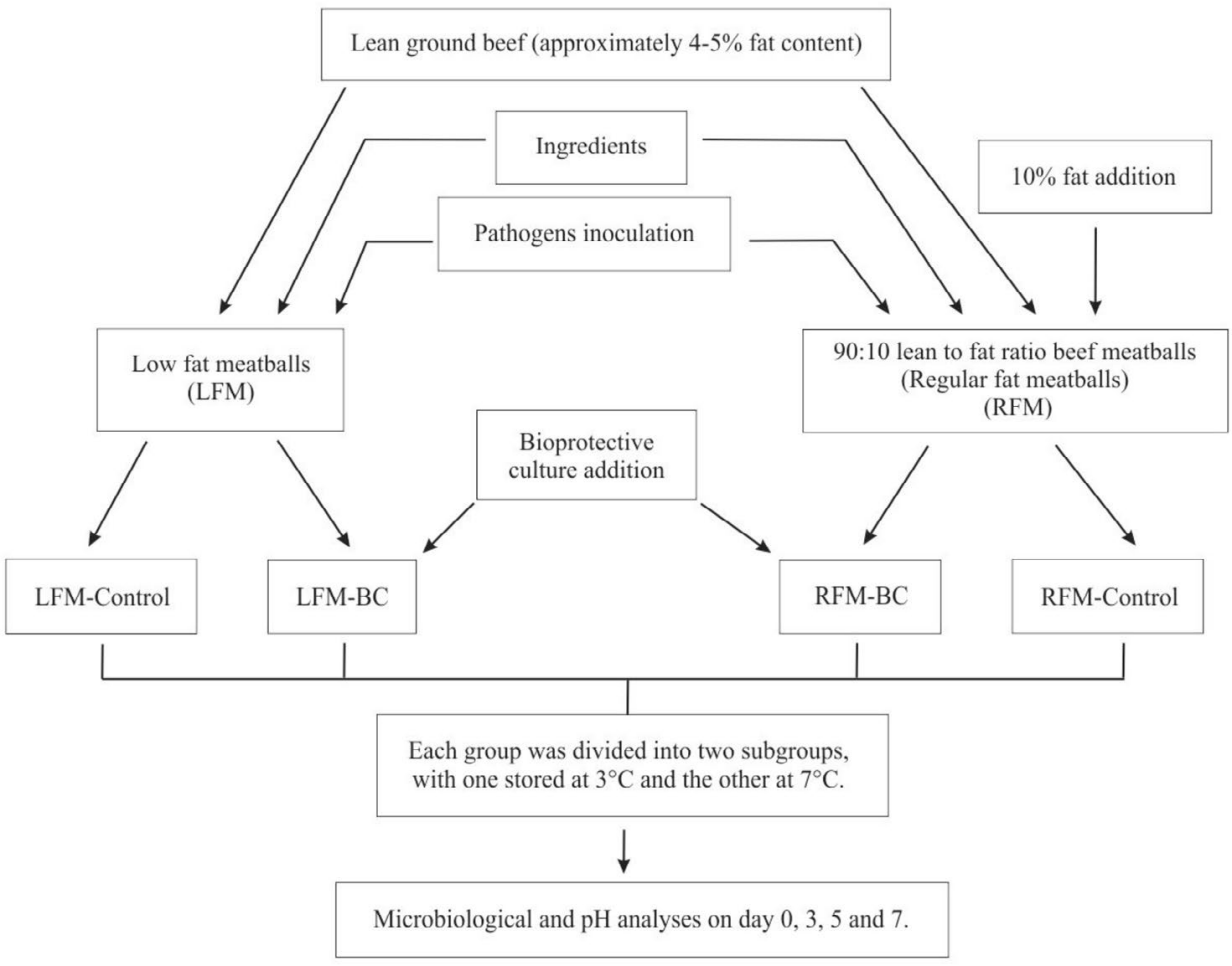
Schematic diagram of the preparation of beef meatballs groups and the analyses performed.

The meatball preparation process is schematically illustrated in Figure 1. A 4 kg portion of lean ground beef was divided equally into two groups. Ingredients listed in Table 1 were added, and the low fat (4%–5%) and regular‐fat (12%–13%) meatball doughs were manually mixed using sterile gloves. Each group was then inoculated under aseptic conditions with 2 mL of the mixed culture containing *Salmonella* spp., *L. monocytogenes* , and *E. coli* O157:H7. After uniformly mixed, each designated group was then split further to form two distinct subgroups: one subgroup was left untreated as the control, while the other received 5 mL of bioprotective culture (approximately 10^9^ CFU/mL).

**TABLE 1:** Beef meatball groups and ingredient ratios (%).

Ingredients	Low fat meatballs	Regular fat meatballs
Lean ground beef	92.8	82.8
Extra beef fat	—	10
Dextrose	0.3	0.3
Salt	1.2	1.2
Black pepper powder	0.5	0.5
Grated onion	2.6	2.6
Dried bread crumbs	2.6	2.6

Meatball portions of approximately 50 g were hand‐shaped and individually placed into sterile stomacher bags. The bags were not vacuum sealed or tightly closed; instead, they were loosely folded to protect the samples while allowing storage under aerobic conditions. The experimental groups were defined as follows:

- LFM‐Control: Low fat meatballs without bioprotective culture
- LFM‐BC: Low fat meatballs with bioprotective culture
- RFM‐Control: Regular‐fat meatballs without bioprotective culture
- RFM‐BC: Regular‐fat meatballs with bioprotective culture

Each group was split again into two distinct subgroups, and the meatball samples were immediately transferred to refrigerated storage at either 3°C (cold chain condition) or 7°C (cold chain abuse) following shaping and packaging. On each sampling day (days 0, 3, 5, and 7), two samples were randomly selected from each treatment group for microbiological and pH analyses within each production replicate. The entire experiment was independently repeated three times on separate production days.

### 2.3 Analyses

#### 2.3.1 Fat Content Analysis

Fat content was determined using the Soxhlet extraction method according to Association of Official Analytical Chemists (AOAC 1990).

#### 2.3.2 pH Measurement

Each sample (10 g) was homogenized with 90 mL of sterile distilled water using a stomacher for 2 min, and the pH of the resulting slurry was measured using a digital pH meter (HI 2211, Hanna Instruments, USA).

#### 2.3.3 Microbiological Analyses

Each sample (25 g) was homogenized in 225 mL of sterile Maximum Recovery Diluent (MRD; Merck, Germany) using a stomacher for 2 min. Serial dilutions were prepared, and microbial analyses were conducted as follows:

To assess total LAB populations, the pour plate method on MRS Agar was used, with cultures being incubated at 30°C for a duration of 48 h. *Pseudomonas* spp. were enumerated using spread plating on *Pseudomonas* CFC‐Selective Agar (Merck, Darmstadt, Germany) followed by incubation at 25°C for 48 h. To confirm the identity of the colonies, five were randomly selected and subjected to the oxidase test. Since *Pseudomonas* spp. are known to be oxidase‐positive, final colony counts were adjusted accordingly based on the test results.

For enumeration of pathogens, the following selective agars were used. CT‐supplemented Sorbitol MacConkey (sMAC) agar (Merck, Darmstadt, Germany) for *E. coli* O157:H7, Xylose Lysine Deoxycholate (XLD) Agar (Merck, Darmstadt, Germany) for *Salmonella* spp., and Oxford Listeria Selective Agar with BS003 supplement (Biokar, France) for *L. monocytogenes* . Spread plating was applied, followed by incubation at 35°C for 24–48 h, and typical colonies were counted.

### 2.4 Statistical Analysis

Three independent manufacturing replicates (batches) were carried out to execute the experiment. All data were subjected to statistical computations through the use of SPSS 22 software (IBM SPSS, USA). Prior to statistical modeling, log_10_ CFU/g transformations were applied to the microbiological findings, while the raw values of pH were utilized directly. Prior to model fitting, normality of residuals and homogeneity of variance were assessed using the Shapiro–Wilk and Levene's tests, respectively. A mixed‐effects model fitted by restricted maximum likelihood (REML) was used to evaluate the effects of treatment, storage day, and their interactions. The treatment factor consisted of eight experimental groups representing combinations of fat content, bioprotective culture application, and storage temperature. Treatment group and storage day were included as fixed effects, whereas batch was included as a random effect to account for inherent variability among production replicates and to avoid pseudoreplication. Pairwise comparisons among estimated marginal means were performed using the Bonferroni adjustment. Statistical significance was accepted at *p* < 0.05.

## 3 Results and Discussion

Fat analysis confirmed the intended distinction between the experimental formulations, with measured fat contents of 4.6% ± 0.4% and 12.5% ± 0.5% for the low‐fat and regular‐fat meatballs, respectively (Table 2).

**TABLE 2:** Measured fat content of low‐fat and regular‐fat meatball formulations (%).

Formulation	Fat content (%)
Low‐fat meatballs	4.6 ± 0.4
Regular‐fat meatballs	12.5 ± 0.5

### 3.1 pH

pH behavior was influenced by the experimental treatment and storage time, as indicated by significant group × day interactions (*p* < 0.05). Therefore, changes in pH over storage differed among the experimental groups. Table 3 showed that bioprotective cultures consistently shifted pH toward acidification, whereas storage temperature promoted alkalinization through spoilage metabolism.

**TABLE 3:** The effect of bioprotective culture addition on pH level in lean and regular‐fat meatballs stored at 3°C and 7°C.

Treatment groups	Storage temperatures (°C)	Days			
		0	3	5	7
LFM‐Control	3	5.88 ± 0.01^Ax^	5.86 ± 0.01^ABx^	5.91 ± 0.01^ABx^	5.80 ± 0.02^Dx^
LFM‐BC		5.86 ± 0.05^Ax^	5.88 ± 0.02^Ax^	5.84 ± 0.03^ABCx^	5.63 ± 0.03^Ey^
RFM‐Control		5.75 ± 0.05^Axy^	5.73 ± 0.06^ABxy^	5.69 ± 0.01^CDy^	5.86 ± 0.03^Dx^
RFM‐BC		5.79 ± 0.05^Ax^	5.72 ± 0.02^Bxy^	5.65 ± 0.01^Dy^	5.63 ± 0.05^Ey^
LFM‐Control	7	5.88 ± 0.01^Ayz^	5.78 ± 0.01^ABz^	5.91 ± 0.03^Ayz^	6.10 ± 0.06^BCx^
LFM‐BC		5.86 ± 0.05^Axy^	5.80 ± 0.04^ABy^	5.93 ± 0.02^Axy^	5.95 ± 0.05^CDx^
RFM‐Control		5.75 ± 0.05^Ay^	5.77 ± 0.02^ABy^	5.75 ± 0.03^BCDy^	6.36 ± 0.01^Ax^
RFM‐BC		5.79 ± 0.05^Ay^	5.76 ± 0.04^ABy^	5.62 ± 0.02^Dz^	6.16 ± 0.02^Bx^

*Note:* ^x–z^Different superscript letters within rows indicate differences between storage days (*p* < 0.05). ^A–E^Different capital letters within columns indicate differences among treatment groups for the same day (*p* < 0.05).

Abbreviations: BC, bioprotective culture mixture consisting of *P. acidilactici* , *L. sakei* , and *L. plantarum* ; LFM‐BC, low‐fat (4%–5%) meatballs group with bioprotective culture; LFM‐Control, low‐fat (4%–5%) meatballs group without bioprotective culture; RFM‐BC, regular‐fat (12%–13%) meatballs group with bioprotective culture; RFM‐Control, regular‐fat (12%–13%) meatballs group without bioprotective culture.

At 3°C, meatballs containing bioprotective cultures showed a pronounced acidification curve during storage (*p* < 0.05). This acidification likely resulted from the low‐temperature fermentation of added carbohydrates—specifically dextrose and breadcrumbs—which provide readily utilizable substrates for LAB (Rama et al. 2024). Although numerical changes were modest, their consistency suggests active metabolic acid production even in the absence of substantial LAB proliferation (Table 4).

**TABLE 4:** The effect of bioprotective culture addition on *Lactobacillus* spp. and *Pseudomonas* spp. in lean and regular fat meatballs stored at 3°C and 7°C (Log_10_ CFU/g).

Treatment groups	Storage temperatures (°C)	Days			
		0	3	5	7
		*Lactobacillus* spp.			
LFM‐Control	3	4.3 ± 0.2^B^	4.4 ± 0.4^C^	4.3 ± 0.1^D^	4.5 ± 0.3^D^
LFM‐BC		6.5 ± 0.2^A^	6.4 ± 0.1^AB^	6.4 ± 0.1^B^	6.3 ± 0.2^C^
RFM‐Control		4.2 ± 0.3^Bx^	4.8 ± 0.3^Cy^	5.3 ± 0.2^Cy^	5.2 ± 0.1^Dy^
RFM‐BC		6.6 ± 0.3^A^	6.5 ± 0.1^A^	6.7 ± 0.2^B^	6.6 ± 0.1^BCx^
LFM‐Control	7	4.3 ± 0.2^Bx^	4.9 ± 0.1^Cy^	4.6 ± 0.4^Dxy^	6.2 ± 0.1^Cz^
LFM‐BC		6.5 ± 0.2^Ax^	6.6 ± 0.1^Ax^	6.7 ± 0.2^ABx^	7.4 ± 0.1^Ay^
RFM‐Control		4.2 ± 0.3^Bw^	5.8 ± 0.2^Bx^	6.4 ± 0.2^By^	7.2 ± 0.3^ABz^
RFM‐BC		6.6 ± 0.3^Ax^	6.8 ± 0.1^Axy^	7.3 ± 0.5^Ayz^	7.6 ± 0.1^Az^
		*Pseudomonas* spp.			
LFM‐Control	3	4.7 ± 0.2^Aw^	5.8 ± 0.2^Bx^	7.5 ± 0.2^BCy^	8.5 ± 0.5^Cz^
LFM‐BC		4.7 ± 0.2^Aw^	5.9 ± 0.2^Bx^	7.4 ± 0.5^BCy^	8.8 ± 0.1^BCz^
RFM‐Control		4.5 ± 0.2^Aw^	6.3 ± 0.3^Bx^	7.1 ± 0.2^Cy^	7.7 ± 0.2^Dy^
RFM‐BC		4.7 ± 0.3^Aw^	6.3 ± 0.3^Bx^	6.9 ± 0.2^Cy^	7.3 ± 0.2^Dy^
LFM‐Control	7	4.7 ± 0.2^Aw^	7.3 ± 0.3^Ax^	8.8 ± 0.3^Ay^	9.4 ± 0.3^Bz^
LFM‐BC		4.7 ± 0.2^Aw^	7.2 ± 0.2^Ax^	8.3 ± 0.3^Ay^	9.4 ± 0.2^Bz^
RFM‐Control		4.5 ± 0.2^Aw^	6.3 ± 0.3^Bx^	8.3 ± 0.2^Ay^	9.6 ± 0.1^Az^
RFM‐BC		4.7 ± 0.3^Aw^	6.3 ± 0.4^Bx^	8.1 ± 0.2^ABy^	9.4 ± 0.4^Bz^

*Note:* ^w–z^Different superscript letters within rows indicate differences between storage days (*p* < 0.05). ^A–D^Different capital letters within columns indicate differences among treatment groups for the same day (*p* < 0.05).

Abbreviations: BC, bioprotective culture mixture consisting of *P. acidilactici* , *L. sakei* , and *L. plantarum* ; LFM‐BC, low‐fat (4%–5%) meatballs group with bioprotective culture; LFM‐Control, low‐fat (4%–5%) meatballs group without bioprotective culture; RFM‐BC, regular‐fat (12%–13%) meatballs group with bioprotective culture; RFM‐Control, regular‐fat (12%–13%) meatballs group without bioprotective culture.

At 7°C, all groups exhibited a substantial pH increase (*p* < 0.05), attributable to the accumulation of basic nitrogenous compounds generated by proteolytic spoilage bacteria (Kolbeck et al. 2021). This effect was most pronounced in samples where *Pseudomonas* spp. reached high populations (Table 4), supporting the role of spoilage microbiota in protein degradation and the production of alkaline metabolites such as ammonia and biogenic amines. The increase was particularly evident in the control groups, where the absence of bioprotective cultures likely allowed more extensive spoilage activity, whereas the pH increase was significantly smaller in the bioprotective groups (*p* < 0.05), indicating that LAB partially inhibited spoilage metabolism even under temperature abuse conditions.

Fat content also influenced pH dynamics. Regular‐fat formulations tended to exhibit slightly lower pH values during early storage. Although the differences were biologically small, they may reflect the lower buffering capacity of lipid‐rich matrices (Toomik et al. 2024) and potential lipid hydrolysis releasing short‐chain fatty acids (Wang et al. 2022; Song et al. 2022). Nonetheless, these effects were secondary to the dominant roles of temperature and LAB supplementation.

Overall, Table 3 demonstrates that bioprotective cultures drive the pH profile toward acidification and slow pH rise under elevated temperatures. These findings indicate that LAB‐based protective cultures can contribute to the stabilization of physicochemical quality by mitigating spoilage‐associated pH increases, particularly when proper refrigeration is maintained.

### 3.2 Lactic Acid Bacteria

As expected, bioprotective cultures established an initial population around 6.5 log_10_ CFU/g and maintained relatively stable counts at 3°C, whereas control groups exhibited only minimal increases (Table 4). This stability suggests that the added strains were well adapted to refrigerated conditions and capable of maintaining metabolic activity without requiring substantial growth.

At 7°C, LAB counts increased in all groups, with a markedly greater rise in regular‐fat samples. This observation is consistent with the notion that lipid‐rich matrices provide more favorable microenvironments, possibly via partial oxygen exclusion or membrane protective effects, which may explain why indigenous LAB proliferated more rapidly (Fonseca et al. 2019; Toomik et al. 2024). Despite this, their counts remained below the levels maintained by the bioprotective cultures.

Despite the differences observed among the experimental groups, the most important pattern was that bioprotective cultures consistently achieved and maintained a numerical advantage throughout storage. This dominance is critical because LAB do not need to reach exponential growth to exert antimicrobial action (Hoyle et al. 2009); the stable counts at 3°C suggest that even without additional proliferation, the protective cultures remained metabolically active enough to acidify the environment or produce inhibitory metabolites.

Collectively, the LAB data suggest that bioprotective cultures can maintain ecological stability in meatballs under both ideal and mildly abusive storage conditions. Fat affected the growth of the indigenous LAB population, but the protective cultures remained dominant regardless of fat level.

### 3.3 *Pseudomonas* spp.

*Pseudomonas* spp. constitute a major group of psychrotrophic microorganisms heavily implicated in the deterioration of meat products under aerobic and refrigerated conditions (Marcelli et al. 2024). Although the standard limit for total aerobic colony counts in meat products is 7.0 log_10_ CFU/g (ICMSF 2002), this study focused specifically on *Pseudomonas* spp. due to the intentional addition of pathogens and bioprotective cultures. A significant group × day interaction was observed (*p* < 0.001), indicating that changes in *Pseudomonas* counts over storage differed among the experimental groups.

At 3°C, regular‐fat meatballs consistently exhibited slower *Pseudomonas* growth than low‐fat formulations (*p* < 0.05). These findings suggest that the slower growth of *Pseudomonas* spp. in regular‐fat meatballs may reflect matrix‐related differences associated with fat content; however, the underlying mechanisms were not investigated in the present study. Previous studies have also reported variations in spoilage behavior in meat systems with different fat contents (Toomik et al. 2024; Valerio et al. 2020), although the factors responsible for these differences are not yet fully understood.

In contrast, the addition of bioprotective cultures did not produce significant inhibitory effects on *Pseudomonas* spp., particularly at 7°C (*p > 0.05)*. This is expected given that LAB typically exert limited antagonism toward Gram‐negative, aerobic spoilage organisms (Xu et al. 2022; Göçmez and İlhak 2025). The nearly identical growth trends between bioprotective cultures and control samples at both temperatures support this conclusion.

In samples stored at both 3°C and 7°C, differences associated with fat content appeared more pronounced than those associated with bioprotective culture supplementation. Although *Pseudomonas* populations exceeded 7 log_10_ CFU/g by day 5 and the sensory shelf‐life was effectively exceeded, analyses were extended to day 7 in order to better characterize microbial interactions and competitive dynamics under controlled experimental conditions. At 7°C, the fat effect diminished; both LFM and RFM groups reached similarly high spoilage loads by day 7 (Table 4). This suggests that once the temperature favors rapid psychrotrophic metabolism, the moderating influence of fat becomes negligible. In contrast, at proper refrigeration temperatures (3°C), fat content retained its protective effect by slowing *Pseudomonas* growth compared to lean formulations.

Despite the observed differences among treatments, the inhibitory effect of the bioprotective culture on *Pseudomonas* spp. remained relatively limited. This finding is consistent with the ecological characteristics of *Pseudomonas*, which is a highly competitive aerobic spoilage organism frequently dominating the microbiota of refrigerated meat products (Zhang et al. 2024). In addition, as a Gram‐negative bacterium, *Pseudomonas* possesses an outer membrane that can reduce susceptibility to many bacteriocins produced by lactic acid bacteria (Li et al. 2018), whose activity is often more pronounced against Gram‐positive microorganisms. Consequently, while bioprotective cultures may contribute to pathogen control and improve microbial safety, their impact on shelf‐life extension may be constrained when spoilage is primarily driven by *Pseudomonas* spp.

In general, the results indicate that *Pseudomonas*‐induced spoilage is primarily determined by temperature, that fat content provides only limited protection under appropriate refrigeration conditions, and that the protective cultures used in the study cannot compensate for inadequate cold chain conditions.

### 3.4 *Escherichia coli* O157:H7

Table 5 showed a consistent inhibitory effect of bioprotective cultures on *E. coli* O157:H7 across both fat levels and temperatures. Throughout storage, *E. coli* O157:H7 counts exhibited a consistent, albeit modest, decline (*p < 0.05*) . Groups containing the bioprotective culture consistently maintained lower pathogen levels compared to the control groups. Notably, fat content did not alter the survival pattern of *E. coli* O157:H7, indicating that, within the tested range, *E. coli* O157:H7 was largely indifferent to variations in lipid level.

**TABLE 5:** The effect of bioprotective culture addition on *E. coli* O157:H7, *Salmonella* spp., and *L. monocytogenes* in lean and regular fat meatballs stored at 3°C and 7°C (Log_10_ CFU/g).

Treatment groups	Storage temperatures (°C)	Days			
		0	3	5	7
		*Escherichia coli* O157:H7			
LFM‐Control	3	4.2 ± 0.3^Ax^	3.6 ± 0.4^ABCy^	3.0 ± 0.3^Ay^	3.2 ± 0.3^Ay^
LFM‐BC		4.1 ± 0.2^Ax^	3.2 ± 0.3^ABCy^	2.7 ± 0.2^AByz^	2.5 ± 0.2^ABCz^
RFM‐Control		4.3 ± 0.3^Ax^	3.6 ± 0.4^ABCy^	3.3 ± 0.3^Ay^	3.1 ± 0.4^ABy^
RFM‐BC		4.3 ± 0.3^Ax^	3.1 ± 0.1^Cy^	3.1 ± 0.4^Ay^	2.4 ± 0.2^BCz^
LFM‐Control	7	4.2 ± 0.3^Ax^	3.8 ± 0.2^ABxy^	3.3 ± 0.2^Ayz^	3.2 ± 0.2^Az^
LFM‐BC		4.1 ± 0.2^Ax^	3.9 ± 0.2^Ax^	2.3 ± 0.3^By^	2.2 ± 0.3^Cy^
RFM‐Control		4.3 ± 0.3^Ax^	3.7 ± 0.4^ABCy^	3.1 ± 0.4^Ayz^	3.0 ± 0.3^ABz^
RFM‐BC		4.3 ± 0.3^Ax^	3.1 ± 0.1^Cy^	2.6 ± 0.2^AByz^	2.5 ± 0.2^BCz^
		*Salmonella* spp.			
LFM‐Control	3	4.1 ± 0.3^Ax^	4.0 ± 0.4^ABx^	3.6 ± 0.1^ABxy^	3.1 ± 0.1^BCy^
LFM‐BC		4.0 ± 0.2^Ax^	3.4 ± 0.1^By^	3.2 ± 0.1^By^	3.0 ± 0.3^BCy^
RFM‐Control		4.3 ± 0.4^Ax^	4.2 ± 0.3^Axy^	3.7 ± 0.2^ABxy^	3.6 ± 0.2^ABy^
RFM‐BC		4.2 ± 0.3^Ax^	3.7 ± 0.3^ABxy^	3.2 ± 0.3^Byz^	2.6 ± 0.2^Cz^
LFM‐Control	7	4.1 ± 0.3^Ax^	4.0 ± 0.3^ABx^	3.7 ± 0.2^ABx^	3.9 ± 0.4^Ax^
LFM‐BC		4.0 ± 0.2^Ax^	3.5 ± 0.4^ABxy^	3.3 ± 0.4^ABy^	3.4 ± 0.2^ABy^
RFM‐Control		4.3 ± 0.4^Ax^	4.3 ± 0.3^Ax^	4.0 ± 0.2^Ax^	3.9 ± 0.1^Ax^
RFM‐BC		4.2 ± 0.3^Ax^	3.7 ± 0.4^ABx^	3.7 ± 0.4^ABx^	1.8 ± 0.3^Dy^
		*Listeria monocytogenes*			
LFM‐Control	3	4.9 ± 0.2^Ax^	4.7 ± 0.1^Ax^	4.8 ± 0.2^ABx^	4.8 ± 0.2^ABx^
LFM‐BC		4.9 ± 0.4^Ax^	4.9 ± 0.1^Ax^	4.5 ± 0.3^ABCx^	4.4 ± 0.4^ABCx^
RFM‐Control		4.7 ± 0.3^Ax^	4.2 ± 0.2^ABCxy^	4.2 ± 0.3^BCDy^	3.8 ± 0.3^DEy^
RFM‐BC		4.7 ± 0.2^Ax^	3.8 ± 0.3^Cy^	3.8 ± 0.3^Dy^	3.2 ± 0.2^Ez^
LFM‐Control	7	4.9 ± 0.2^Ax^	4.8 ± 0.1^Ax^	4.9 ± 0.2^Ax^	5.1 ± 0.2^Ax^
LFM‐BC		4.9 ± 0.4^Ax^	4.5 ± 0.1^ABxy^	4.4 ± 0.3^ABCDxy^	4.2 ± 0.2^BCDy^
RFM‐Control		4.7 ± 0.3^Ax^	4.4 ± 0.1^ABCxy^	4.1 ± 0.3^BCDy^	4.4 ± 0.2^BCDxy^
RFM‐BC		4.7 ± 0.2^Ax^	4.0 ± 0.3^BCy^	4.1 ± 0.2^CDy^	4.0 ± 0.1^CDy^

*Note:* ^x–z^Different superscript letters within rows indicate differences between storage days (*p* < 0.05). ^A–E^Different capital letters within columns indicate differences among treatment groups for the same day (*p* < 0.05).

Abbreviations: BC, bioprotective culture mixture consisting of *P. acidilactici* , *L. sakei* , and *L. plantarum* ; LFM‐BC, low‐fat (4%–5%) meatballs group with bioprotective culture; LFM‐Control, low‐fat (4%–5%) meatballs group without bioprotective culture; RFM‐BC, regular‐fat (12%–13%) meatballs group with bioprotective culture; RFM‐Control, regular‐fat (12%–13%) meatballs group without bioprotective culture.

The suppression observed in bioprotective culture groups likely reflects the combined effects of mild acidification and the presence of LAB. Even at 3°C, where LAB proliferation was limited, stable LAB populations appeared sufficient to maintain low level metabolic activity and generate an inhibitory environment. Previous studies similarly reported that protective cultures can reduce *E. coli* O157:H7 viability without requiring extensive growth, consistent with a metabolite‐driven rather than competitive exclusion mechanism (Hoyle et al. 2009; Prazdnova et al. 2022; Baillo et al. 2023). At 7°C, the survival of *E. coli* O157:H7 was not markedly different from that at 3°C, suggesting that within this moderate storage temperature range, the pathogen's physiology remains same. However, bioprotective cultures still provided a measurable inhibitory effect.

In general, although the reductions are moderate, the consistent suppression of *E. coli* O157:H7 under all conditions indicates that bioprotective cultures can act as a supportive barrier against *E. coli* O157:H7. Although not sufficient as a single intervention, when combined with adequate cooling, they can make a meaningful contribution to multi‐barrier technology.

### 3.5 *Salmonella* spp.

The survival pattern of *Salmonella* spp. showed greater variability across formulations and storage conditions than that observed for *E. coli* O157:H7. As shown in Table 5, a significant difference among experimental groups was observed on day 7 (*p* < 0.05). Regular‐fat meatballs containing bioprotective cultures exhibited the most pronounced reductions, particularly under temperature abuse conditions, suggesting that the combined effects of product formulation and storage conditions may influence antimicrobial effectiveness. Similar to *E. coli* O157:H7, changes in *Salmonella* levels occurred alongside increased LAB activity and rising populations of spoilage microorganisms such as *Pseudomonas* spp., indicating a shared response to evolving microbial competition rather than a pathogen‐specific mechanism. Importantly, although reductions in pathogen levels coincided with increased *Pseudomonas* populations, this should not be interpreted as an extension of product shelf‐life. Rather, the observed reductions in pathogens reflect intensified microbial competition under the experimental conditions rather than an improvement in overall product quality or storability.

A plausible explanation is that *Salmonella* may be more susceptible to combined environmental stresses, including mild acidification and intensified microbial competition. Such sensitivity has been previously associated with the combined effects of acid stress and competitive microbial interactions in complex food ecosystems (Mario 2023). The observed reductions in the RFM control groups (approximately 0.4–0.7 log_10_ CFU/g) indicate that fat content alone did not exert a strong antimicrobial effect. However, when bioprotective cultures were present, reductions increased to approximately 1.6–2.4 log_10_ CFU/g, suggesting that matrix composition may modulate the effectiveness of microbial antagonism rather than acting as a direct inhibitory factor.

Temperature appeared to play an important role in shaping *Salmonella* dynamics. At 3°C, reductions were more gradual and seemed to be primarily associated with LAB‐mediated environmental changes, whereas at 7°C, increased microbial activity and competition from rapidly growing spoilage flora may have contributed to greater pressure on *Salmonella* populations. This interpretation is consistent with reports indicating that *Salmonella* can be less competitive than psychrotrophic background microbiota under aerobic, nutrient‐limited conditions. For example, Hoyle et al. (2009) reported that although LAB did not markedly increase in number at 5°C, their presence was associated with reductions of up to 6 log_10_ CFU/g in *Salmonella* spp.

The pronounced reductions observed for *Salmonella* spp. may be related to several complementary mechanisms associated with the bioprotective culture mixture. Lactic acid bacteria are known to suppress *Salmonella* through competitive exclusion, nutrient depletion, and the production of antimicrobial metabolites such as organic acids and bacteriocins (Zapaśnik et al. 2022). In the present study, the combined presence of *P. acidilactici* , *L. sakei* and *L. plantarum* may have contributed to enhanced antimicrobial pressure through the production of inhibitory compounds and ecological competition. Moreover, exposure to refrigerated storage and matrix‐related stresses may have placed *Salmonella* cells under sublethal stress, increasing their susceptibility to antagonistic interactions.

From a food safety perspective, however, the observed reductions should be interpreted with caution. Although the reductions achieved in the present study were statistically significant and reached up to approximately 2.4 log_10_ CFU/g in regular‐fat meatballs supplemented with bioprotective cultures, they remain below the 5‐log reductions commonly associated with validated pathogen control processes. Therefore, the protective cultures evaluated in this study should not be considered a standalone intervention for *Salmonella* control. Rather, their practical value lies in their use as an additional hurdle that complements refrigeration and other food safety measures within a multi‐barrier preservation strategy.

Overall, these findings suggest that bioprotective cultures can meaningfully suppress *Salmonella* spp., particularly in regular‐fat formulations and under mild temperature abuse. This underscores the potential role of matrix composition and storage conditions in shaping pathogen behavior.

### 3.6 *Listeria monocytogenes*

The behavior of *L. monocytogenes* differed from that of the other tested pathogens, with reductions varying among the experimental groups and storage conditions. As shown in Table 5, significant differences among treatment groups were observed over storage (*p* < 0.05), indicating that the response of *L. monocytogenes* was influenced by differences in formulation and storage conditions. In low‐fat formulations, *L. monocytogenes* populations remained relatively stable throughout storage, particularly at 3°C, suggesting limited inhibitory pressure under these conditions. In contrast, in regular‐fat meatballs, a gradual but consistent decline was observed, with reductions becoming more pronounced toward the end of storage. This trend was particularly evident in the RFM‐BC group, where reductions were consistently greater than those observed in the corresponding control groups, indicating an enhanced suppressive effect under high‐fat conditions.

The stronger reductions observed in regular‐fat samples may be linked to alterations in the microenvironment associated with higher lipid content. Increased fat levels can reduce water activity and modify nutrient diffusion, potentially limiting pathogen access to essential substrates (Purk et al. 2023). In line with this interpretation, Baka et al. (2016) reported that increasing fat levels (0%–26%) in frankfurter sausages resulted in reduced proliferation of *L. monocytogenes* . Moreover, lipid‐rich matrices may favor localized accumulation of inhibitory metabolites produced by lactic acid bacteria, thereby intensifying antimicrobial pressure. Although *L. monocytogenes* is known for its tolerance to refrigeration and mild stress conditions (Bucur et al. 2018), the combined influence of fat‐mediated matrix effects and microbial competition appears to have constrained its survival.

In several treatment groups, reductions tended to be greater at 3°C than at 7°C. At 3°C, reductions progressed gradually, suggesting that inhibitory effects were primarily driven by long‐term ecological interactions rather than rapid metabolic suppression. In contrast, at 7°C, reductions were more limited, despite increased overall microbial activity, indicating that higher metabolic activity alone was insufficient to enhance suppression of *L. monocytogenes* . These results suggest that bioprotective cultures may exert stronger inhibitory effects in high‐fat environments under low‐temperature conditions.

Although bacteriocin‐producing lactic acid bacteria are frequently reported to exhibit strong anti‐Listeria activity (Zapaśnik et al. 2022), the reductions observed for *L. monocytogenes* in the present study were generally less pronounced than those recorded for *Salmonella* spp. This apparent discrepancy may reflect the multifactorial nature of bioprotection in complex food matrices. In addition to bacteriocin production, inhibitory effects may involve competition for nutrients, organic acid production, and interactions with storage conditions. Furthermore, matrix‐related factors may limit the diffusion or activity of antimicrobial compounds in meat products, resulting in pathogen responses that differ from those observed in simplified laboratory systems. The comparatively lower susceptibility of *L. monocytogenes* may also be related to its psychrotrophic nature and ability to adapt to acidic and refrigerated environments (Yang et al. 2024). Previous studies have reported that *Listeria* can maintain metabolic activity under cold storage conditions where the growth of competing microorganisms is restricted, thereby reducing the effectiveness of certain bioprotective cultures (Fliss et al. 2025; Engstrom et al. 2021).

Overall, these findings indicate that while *L. monocytogenes* exhibits notable resilience, its survival can be meaningfully reduced when bioprotective cultures are applied within a favorable matrix context. The results underscore the importance of considering both formulation and storage conditions when designing strategies to control this pathogen in meat products.

From a regulatory perspective, the findings of this study are relevant to current food safety frameworks that emphasize preventive and multi‐hurdle approaches to pathogen control. Although the reductions achieved by bioprotective cultures do not replace validated pathogen elimination processes, they may support compliance with microbiological food safety objectives by limiting pathogen survival during refrigerated storage. This concept is consistent with the principles outlined in Commission Regulation (EC) No 2073/2005 (European Commission [EC] 2005) on microbiological criteria for foodstuffs and the Principles and Guidelines for the Establishment and Application of Microbiological Criteria Related to Foods (Codex Alimentarius Commission [CAC] 1997), both of which recognize the importance of preventive measures and process control throughout the food chain.

Despite these promising findings, certain limitations of the present study should be acknowledged. The meatball formulations contained onion, black pepper, and bread crumbs in order to reflect commercially relevant product formulations. Although these ingredients were kept constant across all treatments, some of them may possess antimicrobial properties and could therefore have contributed to the overall microbial responses observed during storage. The present study was designed to evaluate the combined performance of bioprotective cultures within a realistic meatball matrix rather than to isolate the effects of individual ingredients. Future studies using simplified formulations may help clarify potential interactions between formulation components and bioprotective cultures.

## 4 Conclusion

The results demonstrate that bioprotective cultures can contribute to microbial safety in beef meatballs during refrigerated storage by reducing the survival of foodborne pathogens, while exerting only limited effects on spoilage microbiota. The greatest inhibitory effects were observed against *Salmonella* spp. and *E. coli* O157:H7, with reductions of up to approximately 2.4 log_10_ CFU/g under the most favorable conditions, particularly in regular‐fat formulations. In contrast, the response of *L. monocytogenes* was more dependent on matrix composition, with bioprotective cultures generally showing greater effectiveness in regular‐fat than in low‐fat meatballs. In addition, the slower growth of *Pseudomonas* spp. observed in regular‐fat meatballs stored at 3°C suggests that formulation characteristics may influence spoilage dynamics during refrigerated storage.

Overall, these findings highlight the importance of considering both product formulation and storage conditions when designing bioprotective strategies for meat products. However, the magnitude of pathogen reductions observed in the present study indicates that bioprotective cultures should be regarded as a complementary rather than a standalone food safety intervention. Their application may support hurdle‐based preservation strategies aimed at improving microbial safety and microbiological stability in refrigerated meat products. Future studies should further explore strain‐specific interactions, matrix‐dependent mechanisms, and the development of predictive models to better describe microbial responses and optimize the application of bioprotective cultures in industrial meat systems.

## Author Contributions

**Enise Begüm Göçmez:** writing – original draft, writing – review and editing, formal analysis. **Tevhide Elif Güner:** writing – review and editing, writing – original draft. **Osman İrfan İlhak:** project administration, writing – review and editing, writing – original draft, resources, supervision. **Hakan Tavsanli:** writing – review and editing, supervision, formal analysis, writing – original draft.

## Funding

This work is supported by the Scientific Research Project Fund of Balikesir University under the project number 2021/130.

## Disclosure

Author Approval and Responsibility Statement: All authors have read and approved the final version of the manuscript. The Corresponding Author had full access to all of the data in this study and takes complete responsibility for the integrity of the data and the accuracy of the data analysis.

## Ethics Statement

This study was conducted entirely in vitro and in a laboratory environment utilizing commercial bacterial starter cultures and commercial ground beef and fat. The research did not involve any human participants, clinical trials, tissue sampling, or experiments on live animals. Consequently, institutional ethical approval or clearance was not required for this study.

## Conflicts of Interest

The authors declare no conflicts of interest.

## Data Availability

The authors confirm that the data supporting the findings of this study are available within the article.
